# Genome Sequence of *Staphylococcus aureus* Strain CA-347, a USA600 Methicillin-Resistant Isolate

**DOI:** 10.1128/genomeA.00517-13

**Published:** 2013-07-25

**Authors:** Marc Stegger, Elizabeth M. Driebe, Chandler Roe, Darrin Lemmer, Jolene R. Bowers, David M. Engelthaler, Paul Keim, Paal S. Andersen

**Affiliations:** Statens Serum Institut, Department of Microbiological Surveillance and Research, Copenhagen, Denmarka; Translational Genomics Research Institute (TGen), Pathogen Genomics Division, Flagstaff, Arizona, USAb

## Abstract

The *Staphylococcus aureus* clonal lineage CC45 is a predominant colonizer of healthy individuals in northern Europe and constitutes a highly basal cluster of the *S. aureus* population. Here, we report the complete genome sequence of *S. aureus* strain CA-347 (NRS648), a representative of the methicillin-resistant USA600 clone predominantly found in the United States.

## GENOME ANNOUNCEMENT

In Europe and the United States, the *Staphylococcus aureus* clonal lineage CC45 isolates are predominantly found in the nasal community of healthy individuals (1–7) and in large numbers in bloodstream infections (8, 9). There are two major geographically distinct sublineages of methicillin-resistant *S. aureus* (MRSA) CC45 found primarily in the United States (USA600) or in central Europe (Berlin-IV). Here, we present the complete genome sequence of a USA600 representative isolate, *S. aureus* strain CA-347, from a bacteremia infection in 2005 in California.

CA-347 was obtained from the Network on Antimicrobial Resistance in *Staphylococcus aureus* (NARSA) (http://www.narsa.net; accession no. NRS648). The genome sequence was acquired using 179 Mb of paired-end reads (101-bp reads, 400-bp spacing) and 502 Mb of mate-pair reads (100-bp reads, 5-kb spacing) from Illumina platforms (Illumina, San Diego, CA) and by using data from four PacBio RS SMRT cell runs (Pacific Biosciences, CA) that generated ~200,000 reads (298 Mb of sequencing data), with an average sequence length of ~1,500 bp, and with 2,824 reads of >6 kb. The raw PacBio sequence reads were error corrected with 50× Illumina paired-end data and PacBio CCS data using the PacBioToCA tool (10). The data were assembled using the Celera assembler (10) on long PacBio error-corrected sequences (>6 kb) with 18× coverage, and with assembly of the Illumina paired-end data generated using ABySS v1.3.5 (11), with subsequent contig extension using PBJelly with the error-corrected PacBio reads (12). The Celera assembly provided six contigs with an N_50_ of 2,677 kb, and the ABySS and PBJelly assembly provided 25 contigs with an N_50_ of 419 kb. An optical map (OpGen, MD) of CA-347 was also obtained.

The chromosome was assembled from a combination of the two *de novo* analysis results and by comparative genomics with other *S. aureus* reference genome sequences using Genomics Workbench 6.02 (CLC bio, Aarhus, Denmark), before verification against the optical map. Plasmid pCA-347 was present on a single contig from the Celera assembler. The genome was annotated using the IGS Annotation Engine (http://ae.igs.umaryland.edu/cgi/index.cgi) with manual curation.

The genome consists of a 2,850,503-bp circular chromosome with a G+C content of 32.9%, containing 2,696 coding sequences (CDSs), 60 tRNAs, and 16 rRNA features.

The CA-347 isolate carries a staphylococcal cassette chromosome *mec* element (SCC*mec*) type II, which is highly similar to the SCC*mec* type II found in *S. aureus* strain N315 (13), and two complete prophages, φSa2 and φSa3, as well as two incomplete prophages identified by PHAST analysis (14). Phylogenetic analysis against the major clonal lineages of *S. aureus* showed CC45 to form a distinct basal cluster within the *S. aureus* population. CA-347 contains a 24,653-bp plasmid, pCA-347, with 29 CDSs that is essentially identical to pN315 (13), differentiated by only 36 single nucleotide polymorphisms and three small indels.

The presented genome sequence is the first available complete genome sequence of this clonal complex. Comparative analyses can highlight important properties of *S. aureus* evolution and potentially reveal genetic features associated with commensal carriage of *S. aureus* to explain the high carriage rate of CC45.

### Nucleotide sequence accession numbers.

The complete sequences of the chromosome of *S. aureus* CA-347 and plasmid pCA-347 have been deposited in GenBank under the accession no. CP006044 and CP006045.
